# Development and application of affordable SNP typing approaches to genotype *Mycobacterium tuberculosis* complex strains in low and high burden countries

**DOI:** 10.1038/s41598-019-51326-2

**Published:** 2019-10-25

**Authors:** Irving Cancino-Muñoz, Ana Gil-Brusola, Manuela Torres-Puente, Carla Mariner-Llicer, John Dogba, Victor Akinseye, Kehinde Adesokan, Ayi Kwaghe, Francis Ejeh, Simeon Cadmus, Iñaki Comas

**Affiliations:** 1 Instituto de Biomedicina de Valencia (IBV-CSIC), Valencia, Spain; 2 FISABIO Public Health, Genomics and Health Unit, Valencia, Spain; 3 Hospital Universitari I Politècnic La Fe, Microbiology Department, Valencia, Spain; 4 University of Ibadan, Department of Veterinary Public Health & Preventive Medicine, Ibadan, Nigeria; 5 University of Ibadan, Centre for Control and Prevention of Zoonosis, Ibadan, Nigeria; 6 Federal Ministry of Agriculture and Rural Development, Department of Veterinary and Pest Control Services, Garki, Nigeria; 7 University of Maiduguri, Department of Veterinary Microbiology, Maiduguri, Nigeria; 8 CIBER of Epidemiology and Public Health (CIBERESP), Madrid, Spain

**Keywords:** Molecular evolution, Pathogens, Infectious-disease diagnostics

## Abstract

The *Mycobacterium tuberculosis* complex (MTBC) comprises the species that causes tuberculosis (TB) which affects 10 million people every year. A robust classification of species, lineages, and sub-lineages is important to explore associations with drug resistance, epidemiological patterns or clinical outcomes. We present a rapid and easy-to-follow methodology to classify clinical TB samples into the main MTBC clades. Approaches are based on the identification of lineage and sub-lineage diagnostic SNP using a real-time PCR high resolution melting assay and classic Sanger sequencing from low-concentrated, low quality DNA. Thus, suitable for implementation in middle and low-income countries. Once we validated our molecular procedures, we characterized a total of 491 biological samples from human and cattle hosts, representing countries with different TB burden. Overall, we managed to genotype ~95% of all samples despite coming from unpurified and low-concentrated DNA. Our approach also allowed us to detect zoonotic cases in eight human samples from Nigeria. To conclude, the molecular techniques we have developed, are accurate, discriminative and reproducible. Furthermore, it costs less than other classic typing methods, resulting in an affordable alternative method in TB laboratories.

## Introduction

With around 10 million new cases and 1.5 million deaths, tuberculosis (TB) caused by the acid-fast bacillus *Mycobacterium tuberculosis* complex (MTBC), is the first worldwide infectious disease cause of death and remains a major global health problem in low and high burden incidence countries^1^. The MTBC comprises the species responsible for most of the human TB cases worldwide (*M. tuberculosis* and *M. africanum)* as well as those associated with animal disease (*M. bovis, M. caprae, M. canetti*) and the vaccine strain *M. bovis* BCG^2^. Human-associated strains can be further divided into seven main MTBC lineages^3,4^ using robust genomic markers as single-nucleotide polymorphisms (SNPs) that are in agreement with previous genotyping approaches^5^. Some lineages have been associated with a wide geographic distribution, such as the MTBC lineage (L) 4, which is the most predominant around the globe. On the other hand, other lineages are restricted to certain areas^6^, such as L7 strains predominantly found in Ethiopia^7^, the *M. africanum* L5 and L6, primarily found in West Africa^8^. MTBC L4 is considered the most frequent and genetically diverse. Recently, 10 L4 groups or sub-lineages have been proposed^6^.

Lineages and sub-lineages have been associated with different functional and disease phenotypes including differences in transcription, lipids or immunological response but also with disease presentation and epidemiology^9^. However, it has been difficult to correlate specific nucleotide changes or identify regions associated with those phenotypes. Part of the problem is the complex interaction between the host, the bacteria and the environment^10^. As important as these factors are, the need for robust mycobacterial classification systems that can be used worldwide and compared across sites is sought after. Classical genotyping methods such as Spoligotyping^11^ and the Mycobacterial Interspersed Repetitive Units- Variable Number of Tandem Repeats (MIRU-VNTR)^12^ can fail to discriminate and classify within the MTBC phylogeny^13^. On the contrary, SNP markers are stable over time due to the absence of recombination within MTBC and are congruent with other robust markers like large deletions^4,13^. Other new techniques like Whole-Genome sequencing (WGS) have a greater resolution but are limited by high costs, difficulties to interpret the results and limited access in low-to-middle income countries.

Recently, two new approaches became available by using real-time PCR, and a ligation-dependent PCR with Luminex flow cytometer technology for genotyping clinical MTBC strains^14^. Here, we present two methods developed for fast, accurate and less expensive MTBC genotyping using High Resolution Melting (HRM) analysis with real-time PCR reactions (real-time PCR-HRM) on multiplex and uniplex reactions with an unspecific dye, and automatized Sanger sequencing. HRM analysis assays were performed before on MTBC strains, nevertheless, all studies focused on the detection of variants related with drug resistance^15–18^, and to differentiate MTBC members in cultured and non-cultured samples^19^.

In addition, we applied these new approaches to a collection of 491 clinical uncharacterized isolates from three different burdens countries: (i) human derived samples from a low-burden region in Spain; (ii) human derived samples from a high-burden region in Liberia, West Africa; and (iii) human and cattle derived samples from abattoirs in Nigeria. The three datasets were used to show an accurate picture of the circulating lineages and sub-lineages in the different regions and to explore zoonoses between humans and cattle. Furthermore, we successfully tested our molecular assays in complex biological samples that included low DNA concentrations, unpurified heat-killed extracts, as well as contaminants that could affect the PCR performance. These methods were developed in the need to reduce the costs of typing in diagnostic laboratories, especially in high burden countries.

## Results

### Lineage and sub-lineage identification by real-time PCR-HRM and Sanger sequencing

**Figure 1:**
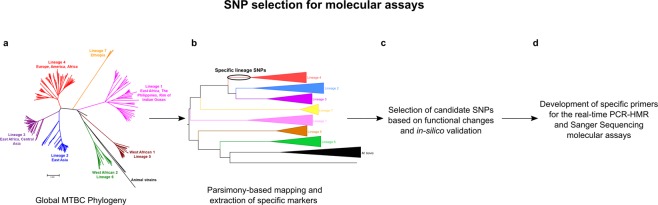
Workflow to identify and select diagnostic lineage/sub-lineage SNP for the development of the molecular assays. (**A**) We used a publicly available variant list containing 34,167 SNPs from 219 MTBC global strains. (**B**) First, we performed a parsimony-based approach to map and obtain 2,056 specific SNPs markers for all lineages, and 1,337 for all L4 sub-lineages. In the figure, the specific SNPs for lineage 4 in the phylogeny are marked with a circle. (**C**) Then, candidate SNPs were selected according to their functional change (we used synonymous SNPs in essential genes, whenever possible), and tested *in-silico* against a global dataset of 4,495 MTCB strains. (**D**) Finally, we developed seven specific primers to implement in the real-time PCR-HRM molecular assay, and two genomic regions for Sanger sequencing approach.

To identify diagnostic SNPs for typing all MTBC lineages and L4 sub-lineages, we analyzed a global reference collection of 219 MTBC strain genomes. A total of 34,167 SNPs were found affecting a variable number of strains as previously published. We mapped the variants to the corresponding MTBC phylogeny and extracted all that were common to the strains belonging to a lineage or sub-lineage. A total of 2,056 lineage SNPs and 1,337 sub-lineage SNPs were identified as candidate markers. Specific common L7 variants were discarded. The diagnostic potential of all variant candidate were corroborated *in-silico* in a larger collection of 4,495 MTBC genomes^20^ to assure their stability (see Methods section and Fig. 1).

Using the annotated specific lineage variant list, we identified two short genomic regions (less than 500 bp) containing SNP markers for the six main human lineages (L1-L6). The first region involved the specific phylogenetic markers for L1, L2 and L5 at H37Rv (NCBI, NC_000962.3) reference genomic positions 4357773GA, 4357804TG, 4357657GA, while the second region included SNPs for L3, L4 and L6 at positions 1281984GA, 1281771CT, 1281685CG, respectively (Supplementary Table 1). After amplifying both regions and testing the specificity of the primers (Supplementary Figs 1 and 2), we used 18 DNA strains representative of the MTBC lineages diversity as controls (reference dataset, Supplementary Table 2) to performed Sanger sequencing. A visual analysis of the sequences generated showed that no nucleotide differences in the amplified regions were detected against the wild type sequences, except for those mutations corresponding to each specific lineage (Supplementary Fig. 3). The diagnostic amplicon positions in the first region for L1, L2 and L5 markers were 243GA, 274GA and 127TG, respectively. In the case of the second region, amplicon positions for detecting L3, L4 and L6 were 310GA, 274GA and 127TG, respectively.

**Figure 2:**
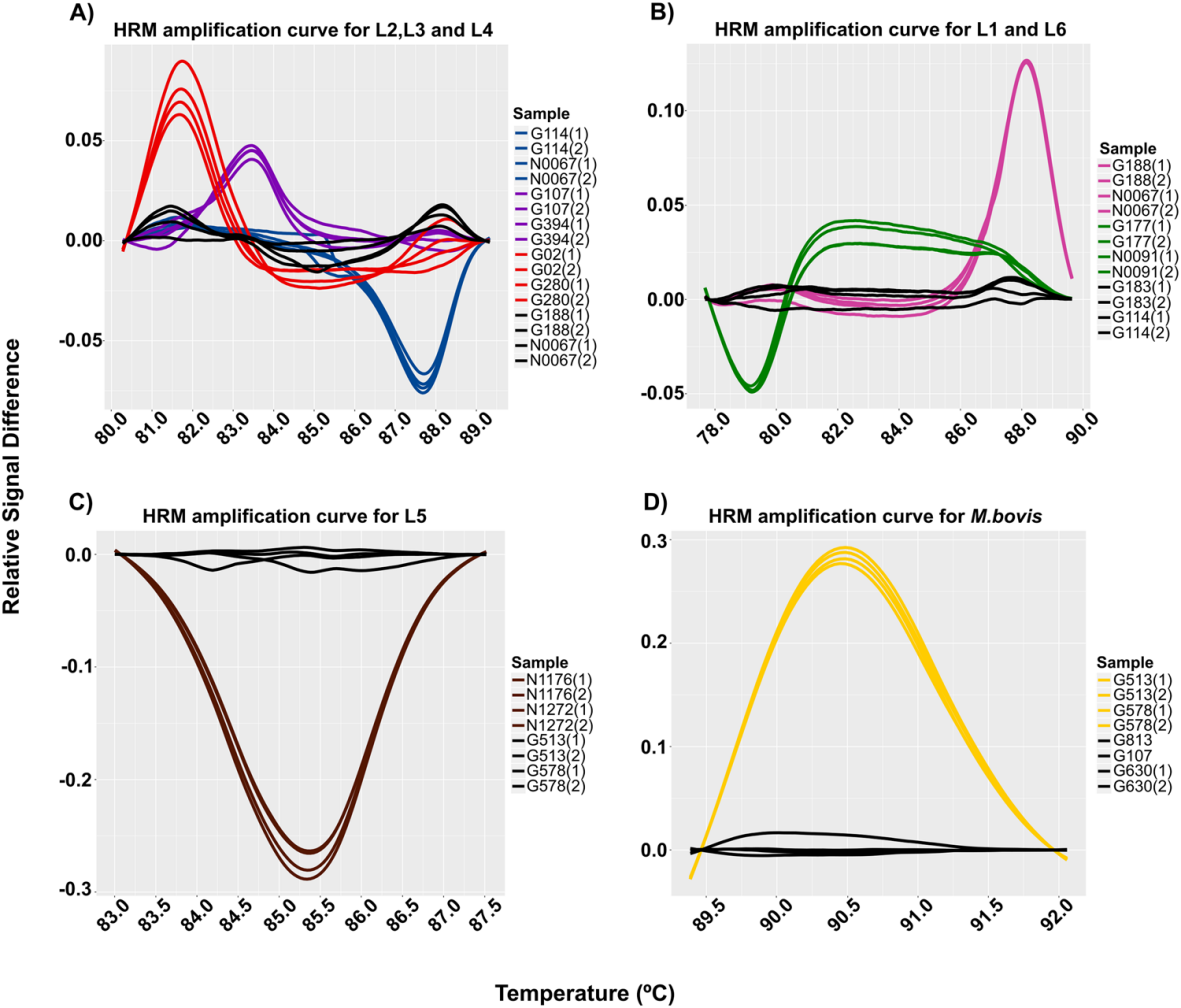
Amplified melting curves for the detection of the MTBC lineages. The graphs show the difference of the melting curves obtained by HRM analysis. Each line indicates a different sample. (**A**,**B**) Curves obtained by Multiplex PCR; (**A**), blue lines represent lineage 2; purple lines represent lineage 3; and red lines indicate lineage 4; (**B**), pink lines represent lineage 1; green lines represent lineage 6. (**C**,**D**) Curves obtained by Uniplex PCR; (**C**), brown lines indicate lineage 5; (**D**), yellow lines represent *M.bovis* lineage. In all cases black lines indicate a wild type genotype.

Different candidate SNP were selected based on different features to design a set of primers for each lineage and sub-lineage for real-time PCR-HRM assay (Tables 1–2**)**. Seven diagnostic SNPs were tested for the real-time PCR-HMR detection of MTBC lineages on the reference dataset (n = 40) (Supplementary Table 2). To optimize the reaction, we developed two multiplex real-time PCR reactions to detect the most common MTBC lineages (L1-L4 and L6). While specific lineages (L5 and *M. bovis*) were detected by a uniplex PCR reaction each, analysis of melting curves on HRM assays denoted the differences between positive controls according to their melting temperature and signal fluorescence (Fig. 2).

**Table 1:** Specific lineage primers used in the study.

MTBC Lineage*	SNP position^†^	Nucleotide change^‡^	Gene	Primers 5′-3′	Amplicon size	Reference
1	115499	T/G	Rv0101	F-ATAATATTGCGTCGGTGTTGG	81 bp	This study
				R-TTATATATTAATGGGCAGGCC		
2	3304966^§^	G/A	Rv2952	F-TGTTACCCGCACTTTCGGCGTTT	80 bp	This study
				R-AGGTCGGCGTATGGGAGGTA		
3	4266647^§^	A/G	Rv3804c	F-GCGACATACCCGTGACGGC	92 bp	This study
				R-CGTTGAGATGAGGATGAGGG		
4	2154724	A/C	Rv1908c	F-CCGAGATTGCCAGCCTTAAG	64 bp	^5^
				R-GAAACTAGCTGTGAGACAGTC		
5	456731	C/T	Rv0380c	F-GCATCGTGTCCGAAGTTCTC	68 bp	This study
				R-ATCATCGCCGACATCGATAC		
6	378404	G/A	Rv0309	F-CCGACAGCGAGAACCTGC	54 bp	^13^
				R-CCATCACGACCGAATGCTT		
*M.bovis*	2831482	T/G	Rv2515c	F-GTGTTGCTGTCGATGACGC	91 bp	This study
				R-ACTGGTACCGCAATACCGTC		

^*^Nomenclature proposed by Comas *et al*.^32^.

^†^Genomic position on the H37Rv reference genome (NCBI, NC_000962.3).

^‡^Allelic change in the reference genome.

^§^Mutation previously described by Fenner *et al*.^33^.

**Table 2:** Specific L4 sub-lineages primers used in the study.

MTBC Sub-lineage*	SNP position^†^	Nucleotide change^‡^	Gene	Primers 5′-3′	Amplicon size	Reference
L4.1.1	3798451^§^	C/G	Rv3383c	F-ATCGACTCAATGGCCCGATG	112 bp	This study
				R-TGACTCTGGATGCGGTTTT		
L4.1.2	4323348^§^	C/T	Rv3848/3849	F-AAATCCGTTCGTCGTGTGGA	82 bp	This study
				R-CTGACGTTGTGAGGGGTCAA		
L4.1.3	4409231	T/G	Rv3921c	F-GACCGCCTCCTGCTTTTTG	53 bp	This study
				R- ACGTCTTCGGCATGATCGAA		
L4.2	2942377	C/T	Rv2614c	F-GAGTAGTCCTCCAGTTCGCG	85 bp	This study
				R-TCAGCTTCCCCGACGAAATC		
L4.3	1480024^§^	G/T	Rv1318c	F-CAGGCCAGGATCCACATCAG	100 bp	This study
				R-TGCTGCTCAATCTCACTCGG		
L4.4	4307886	G/A	Rv3834c	F-AAGGTGGTGCAGTTCGAC	69 bp	This study
				R-ACTGCGAGGCGTGGATTC		
L4.5	2789341^§^	A/C	Rv2483c	F-GGAGGCCTCACCATCCTTG	81 bp	This study
				R-ACGAAGGCGGCTACAAAGAA		
L4.6.1	435708	G/A	Rv0357c	F-CAAAGATCCCGCTGGGTCAT	58 bp	This study
				R-GATATGAGATCGACGGCCGG		
L4.6.2	3191099^§^	C/A	Rv2881c	F-CATCATGCAGAACACCCATC	72 bp	This study
				R-CCCATTGTTCTGCTCTTTCG		
L4.10	1692141^§^	C/A	Rv1501	F-GCTCGGTGTTCTTCGACTCA	107 bp	This study
				R-TGGCCGTTTCAGATAGCACA		

^*^Nomenclature proposed by Stucki *et al*.^6^.

^†^Genomic position on the H37Rv reference genome (NCBI, NC_000962.3).

^‡^Allelic change in the reference genome.

^§^Mutation previously described by Stucki *et al*.^6^.

**Figure 3:**
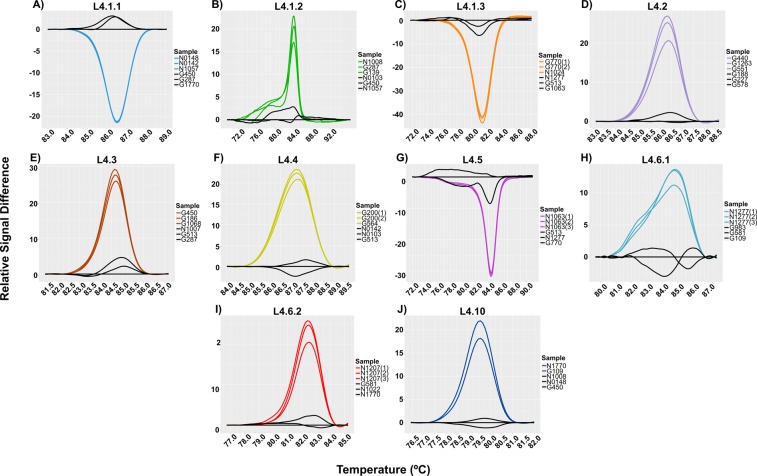
Amplified melting curves for the detection of the MTBC L4 sub-lineages. The graphic shows the difference of the melting curves obtained by HRM analysis. Each letter represent a difference plot of every sub-lineage tested. (**A**) Sub-lineage L4.1.1. (**B**) Sub-lineage L4.1.2. (**C**) Sub-lineage L4.1.3. (**D**) Sub-lineage L4.2. (**E**) Sub-lineage L4.3. (**F**) Sub-lineage L4.4. (**G**) Sub-lineage L4.5. (**H**) Sub-lineage L4.6.1. (**I**) Sub-lineage L4.6.2 and (**J)**. Sub-lineage L4.10. In all cases black lines indicate a wild type genotype.

Given the importance of L4 as the most successful MTBC lineage, we developed a real-time PCR-HRM assay to type the most common sub-lineages defined by WGS. We used six diagnostic SNPs previously described^6^, while the rest were designed after screening of a global SNP database^3^ In all cases, primers were designed to assure the specificity of the real-time PCR-HRM assay (Table 2). Once more, we used 24 DNAs representing the most common L4 sub-lineages (reference collection). HRM analysis showed a clear discrimination between positive samples (those with the specific SNP) against those with wild-type genotype (Fig. 3).

### Performance of typing methodologies

In the previous section we used a reference dataset to set-up the real-time PCR-HRM and Sanger sequencing assays. To evaluate and test their robustness, we calculated some performance parameters from a collection of 76 heat-inactivated clinical samples that had been whole genome sequenced in our laboratory (43 from Liberia dataset and 33 from Valencia), ranging from 0.054–8.08 ng/µl DNA concentrations.In both cases, WGS was use as a gold standard genotyping method.

The sensitivity if the real-time PCR-HRM assay was 97.37% (74/76, [95% CI: 98.82–99.68%]), while the specificity was 100% (368/369, [95% CI: 99.01–100%]).Overall, real-time PCR-HRM assay accuracy was 99.33% (95% CI: 98.04–99.86%).

The sensitivity of the Sanger sequencing was 71.05% (54/76, [95% CI: 59.51–80.89%]), while the specificity was 100% (305/305, [95% CI: 98.80–100.00%]), giving a negative predictive value of 92.44% (95% CI: 89.52–94.56%). Overall, the Sanger Sequencing assay accuracy was 94.23% (95% CI: 91.39–96.35%) (Table 3). Thus, although Sanger sequencing allowed us to accurately identify the lineage, a positive result strongly depends on the successful amplification of at least one genomic region.

**Table 3:** Performance values of the molecular techniques used in the study.

Screening method	No. of samples analyzed (n = 76)	Sensitivity (95% CI)	Specificity (95% CI)	PPV	NPV (95% CI)	Accuracy (95% CI)
Real-time PCR-HRM	74	97.37% (98.82–99.68)	100.00% (99.01–100.00)	100%	99.46% (97.91–99.86)	99.33% (98.04–99.86)
Sanger Sequencing	54	71.05% (59.51–80.89)	100.00% (98.80–100.00)	100%	92.44% (89.52–94.56)	94.23% (91.39–96.35)

These values were extracted from 76 whole-genome sequenced heat-inactivated clinical samples. Abbreviations: PPV, Predicted Positive Value; NPV, Negative Predictive Value.

### Molecular characterization in a low-burden region

**Figure 4:**
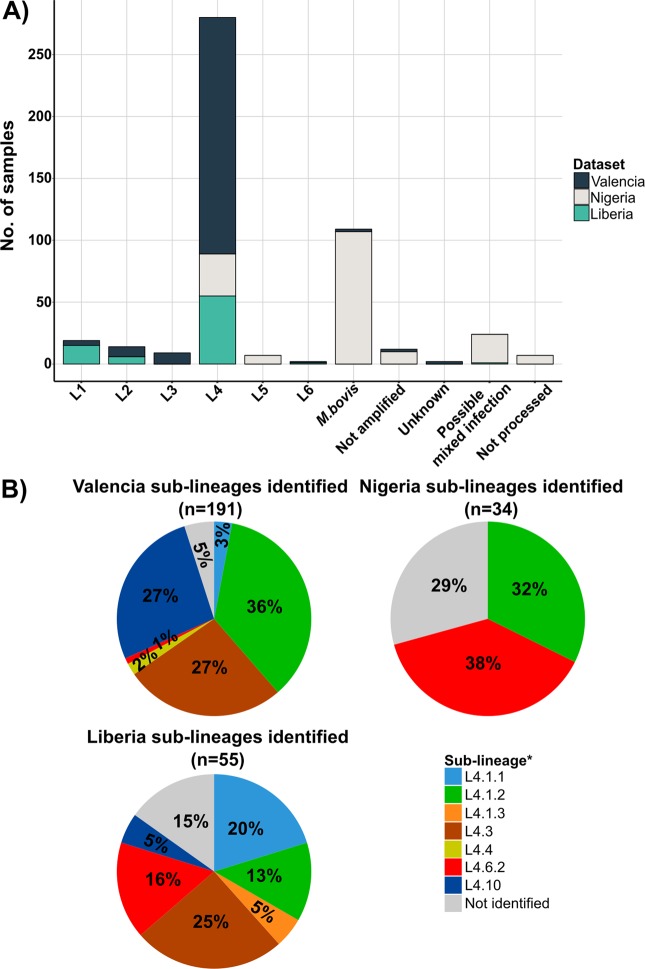
MTBC genotypes identified by real-time PCR-HRM assay in the three study regions. (**A**) Proportion of the main lineages detected. (**B**) Principal sub-lineages belong to L4. Numbers inside of the pie charts represents percentage. *Nomenclature proposed by Stucki *et al*.^6^.

**Figure 5:**
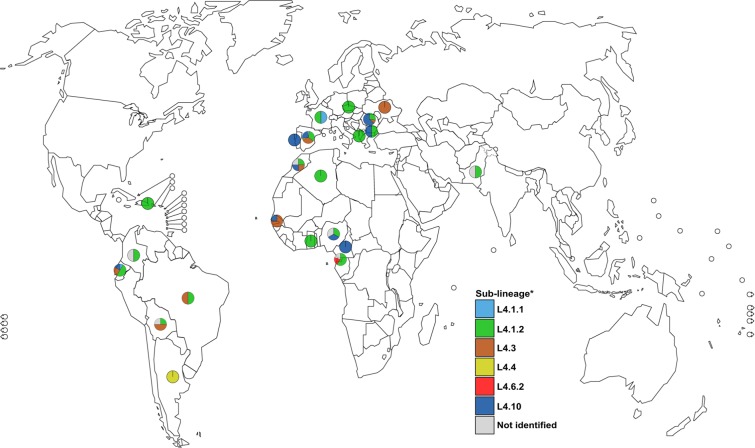
Global distribution of all lineage 4 samples in Valencia region by patient origin country. *Nomenclature proposed by Stucki *et al*.^6^.

Once the methods were optimized and validated, we sought to apply the real-time PCR-HRM technique to heat-inactivated bacteria from a clinical laboratory in order to test the robustness of the typing scheme to DNA amount and purity. We used 219 DNA samples from Valencia (Spain) ranging from 0.02 to 18.3 ng/µl total DNA amount. These samples were heat-inactivated extracts and had not been molecularly characterized before. SNP typing identified 191 isolates (87.2%) as L4, whereas the L3 and L2 were identified in nine (4.1%) and eight (3.6%) samples each. L1 and *M. bovis* lineages were present in four and two cases, respectively. Finally, L5 was not identified. The most frequent sub-lineages identified were L4.1.2 (Haarlem family) with 66 (30%) cases, followed by L4.10 with 51 (23.2%) cases, and L4.3 (LAM family) with 49 (22.3%) isolates (Fig. 4). Furthermore, with the patient origin data obtained from 140 cases, we constructed a map including the sub-lineages frequencies to see whether they are globally spread or just found in restricted areas (Fig. 5). We observed that the sub-lineages L4.1.2 and L4.10 are more frequent in East Europe, while L4.3 is more common in Latin America and East Africa. In contrast, L4.6.2 (Cameroon family) and L4.4 are more specific for some regions of Africa as previously reported^6^.

### Molecular characterization in a high-burden region

We genotyped 266 tuberculosis samples from five different Nigeria districts as well as from Liberia. For this dataset, we also used heat-inactivated extracts. DNA concentrations ranged from 0.05 to 33.2 ng/µl. We were not able to characterize 17 samples due to amplification problems.

**Figure 6:**
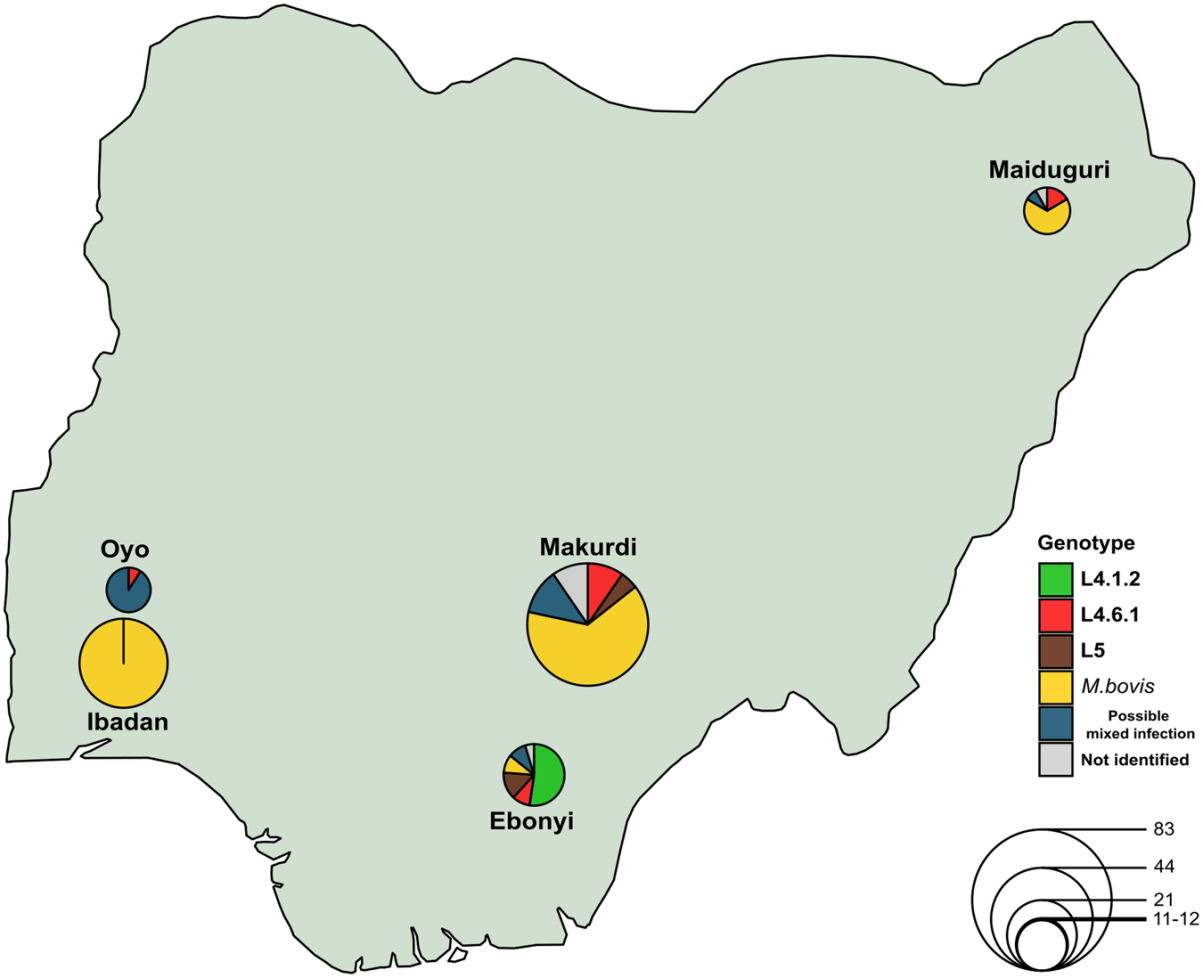
Global distribution of all lineages (sub-lineages included) identified in all Nigeria regions. The map shows the MTBC diversity circulating in Nigeria study regions. The diameter of each circle represents the number of samples obtained from each zone.

The sampling scheme from Nigeria (n = 171) was thought to increase the chances to identify zoonoses events between humans and cattle. Thus 58% of the samples (n = 99) were obtained from cattle lesions while 42% (n = 72) derived from human patients. We screened these samples with our specific *M. bovis* real-time PCR-HRM assay to distinguish between *M. bovis* and the rest of the MTBC strains (Fig. 2D). We detected the presence of an *M. bovis* infection in 99 (93%) cattle lesions. No human tuberculosis was identified in any cattle-derived sample (Table 4). In contrast, we identified bovine tuberculosis infecting eight human cases. We identified that six cases were from Makurdi, North-central-Nigeria. For the rest of the human samples (n = 64), the most common genotype detected was L4 with 34 cases (20%), with the specialist L4.6.2 as the most frequent sub-lineage (13 cases). Surprisingly, we detected the presence of unclassified sub-lineages in 10 samples. L5 was classified in seven isolates. Additionally, ambiguous HRM profiles were identified in 13.5% of the human samples (n = 23). This ambiguous results could be due to the presence of mixed infections by different MTBC strains, potential contamination errors and/or, less likely, to possible PCR artefacts. In addition, with the geographic data, we created a distribution map of the lineages (sub-lineages included), in order to have a snapshot of the MTBC diversity affecting the abattoirs in the zone (Fig. 6).

**Table 4:** MTBC frequencies obtained from Nigeria samples separated by specific host.

MTBC lineage*	Host	
	Cattle	Human
Lineage 4	0 (0%)	34 (20%)
Lineage 5	0 (0%)	7 (4%)
*M.bovis*	99 (58%)	8 (5%)
Possible mixed infection	0 (0%)	23 (13%)
Total	99 (58%)	72 (42%)

^*^Nomenclature proposed by Comas *et al*.^32^.

In the case of the Liberian samples (n = 78), the most frequent lineage detected was L4 corresponding to 70.5% (n = 55) of all MTBC strains, followed by Indio-Oceanic L3 with 19% (n = 15). L2 and L6 were the less frequent lineages with six and one cases, respectively. Within L4, we detected that the L4.3 was the most common with 25.5% of the samples (n = 14), followed by L4.4.1 (X genotype) with 20% of the infecting strains (n = 11). The specific L4.6.2 was found only in nine cases (16.3%) of all the clinical samples (Fig. 4). Furthermore, we were not able to classify eight heat-inactivated L4 MTBC samples, suggesting that local sub-lineages are circulating in the country.

## Discussion

In this study, we developed two genotyping methods using faster and less expensive technologies such as real-time PCR-HRM and Sanger automatized Sequencing with informative SNPs for the main six lineages of MTBC, including the *M. bovis* clade and the most common L4 sub-lineages. First, we set up Multiplex and Uniplex real-time PCR-HRM reactions for the main MTBC lineages and specific L4 sub-lineages using an unspecific dye instead of specific probes, which helps to reduce the cost of the assay. We optimized the PCR assay to obtain a reliable result using lower reagents volumes than the manufacturer’s recommendation (10 vs 20 µl of total reaction volume). By comparing this method with classical genotypic approaches such as spoligotyping which costs ~26US$ per isolate^21^, we demonstrated a relatively lower cost of our assay, being of ~2.4US$ in the case of a uniplex PCR reaction and lower than ~0.9US$ using multiplex condition. Additionally, our molecular techniques have a similar cost when we compared them with other SNP-based genotyping methods such a Luminex MOL-PCR assay, which cost ~0.8 and ~0.15EUR for uniplex and multiplex reactions, respectively^14^. Nevertheless, Luminex platform is considered a not conventional laboratory equipment, especially in low- and middle-income countries. Moreover, we show that our real-time PCR-HRM assay works with heat-inactivated, low-concentrated DNA samples, which are commonly generated in TB diagnostic laboratories.

It is well known that WGS are decreasing every year, mostly based on the high-throughput capacity for sequence several samples per run (up to 24 isolates with the Illumina MiSeq instrument), or because third generation sequencers technologies (such as the Oxford Nanopore MinION device) are more accessible (~100–150EUR per isolate). Besides this, many low- and middle- income countries do not have the necessary equipment. In addition, in many high burden countries, the number of TB cases is larger so it is not feasible to perform WGS all of them^22^, thus providing installations for PCR-based approaches as a quick and affordable screening method.

Using a validation dataset of 76 clinical strains well-defined by WGS to test the performance of the techniques, we were able to genotype up to 97.3% and 71% of samples by real-time PCR-HRM and Sanger sequencing, respectively, both of the with specificity values of 100%. The fact that we could not perform Sanger sequencing assay on some samples, could be due to the low amount of DNA that we were able to recover from them, being insufficient for the minimum required concentration for the amplification on conventional PCR assays. Despite this, all the samples that were amplified by both methods (n = 54), show a 100% of concordance with lineage defined by WGS, even if the techniques differed in the SNP position target. This result suggests that both Sanger sequencing and real-time PCR-HRM assays work with heat-inactivated, and low-concentrated DNA samples, which are commonly generated in TB diagnostic laboratories. We applied our real-time PCR-HRM approach to analyze three uncharacterized collections from different TB burdens countries to test its efficiency. First, we were able to classify 98.6% (215 out of 219) and 93.6% (249 out of 266) of the clinical samples from the low-burden (Valencia, Spain) and high-burden settings (Nigeria and Liberia), respectively. These results are in agreement with studies in which authors mention that up to 6% of the strains were not classified^23,24^. Additionally, we wanted to test the sensitivity of the approach, and we found a positive result while using heat-inactivated clinical samples concentrations below the limit of detection of fluorometric quantitation. These results indicate reliable results, even by using non purified DNA as a template, and as a consequence, a rapid detection method.

Regarding the genotypes identified in Valencia, we found out that majority of the MTBC cases belonged to the global L4 (87%) being subdivided into the generalists L4.1.2 (36%), L4.10 (27.3%), and L4.3 (26.7%). The frequencies detected were in concordance to those reported before worldwide^6^, in the same region^25^, and the same country^26^. Moreover, we identified the rest of the MTBC lineages, except the specific L5, reflecting the high MTBC diversity circulating in the region. Regarding the global distribution of the country of origin of foreign-patients, the analysis shows a congruent result with data reported before. For example, patients infected by L4.10 strains were mostly from East Europe (16 out of 23) also, TB cases from Latin America countries were infected by L4.3 MTBC strains (9 out of 15).

Similar results were obtained with the Liberian samples. We detected that the dominant lineage of the MTBC is L4 (70.5%), followed by L1 (19.2%). As for sub-lineages, we identified the presence of the generalists (L4.3, 25.5%), and specialists (L4.6.2, 16.3%) clades almost with the same frequencies, corresponding with those reported in West African border countries^6,27^. The presence of a high percentage of L4 unclassified samples (14.5%) suggested that endemic sub-lineages (highly likely to be specialist clades) are circulating in the region. This snapshot of the MTBC diversity in the Liberian population denotes the influence of migration inside the country, probably increased after country foundation at the beginning of the 19^th^ century. To our knowledge, this is the first time that Liberian TB samples are genotyped.

The use of a specific lineage marker that identifies the *M. bovis* clade could be helpful to promptly distinguish this genotype from the *M. tuberculosis* strains in clinical samples. As a proof of concept, we performed our real-time PCR-HRM technique on 171 uncharacterized samples mostly from cattle. We found out that the majority of cases (60%, n = 107) belonged to *M. bovis* species. Furthermore, we identified six human-samples harbouring bovine tuberculosis from the same region.

One limitation of these assays is that we only interrogated one specific SNP, and as a consequence, additional biological information such as epidemiological markers (e.g. transmission clusters detection) will be missed. Nevertheless, these techniques are flexible and could be adapted to identify other specific polymorphisms like, for example, the detection of antibiotic resistant MTBC samples^18,28,29^, or the rapid identification of local transmission clusters^23^. We optimized the protocol using the Roche LightCycler480 system which could be an uncommon laboratory device. Nevertheless, we obtained reliable results using the less expensive LightCycler96 instrument. In fact, West African samples were genotyped using this system.

In summary, the molecular approaches developed here show an accurate, discriminative and reproducible methodology to genotype MTBC strains. Due to a need for common and affordable reagents, these techniques could be useful in TB diagnostic laboratories from low- to middle-income countries.

## Methods

### Ethics statement

For the Valencia biological samples, the study was approved by the corresponding Ethics Committee of the Regional Health Office for Valencia (Spain), with an exemption for informed consents from the corresponding Ethics Committee on the basis that this study is part of the surveillance program of communicable diseases by the Public Health Regional Program and, as such, falls outside the mandate of the corresponding Ethics Committee for Biomedical Research. For the Nigerian samples: Based on the premise that the country has a high level of illiteracy, verbal consent was obtained before sample collection. This was approved by the UI/UCH Ethics Committee of the University of Ibadan (UI/EC/14/0198 number). For the Liberian samples: Formal ethical approval for the study was obtained from the Liberian Institute for Medical Research (EC/LIB/914/923 resolution number). Thereafter, the Liberian National Leprosy and Tuberculosis Control Programme and Ministry of Health and Social Welfare approved to collect samples from the hospitals used. For the Liberian samples, there was no direct contact with the patients and therefore exemption from informed consent was granted. All patient personal information was anonymized and no data allowing individual identification was retained. All research was performed following relevant guidelines and regulations.

### Biological samples

We used a reference set of strain DNA samples to set-up the assays^30^. The set included 40 DNA samples corresponding to all MTBC lineages and the main L4 sub-lineages obtained in collaboration with The Swiss Tropical and Public Health Institute (Swiss TPH, [n = 22]), as well as by a local ongoing TB project (n = 18) (Supplementary Table 2). Swiss TPH Reference samples were well defined by WGS in previous studies^3,6^. We used this reference set to optimize the real-time PCR-HRM assay as well as Sanger sequencing. Afterwards, we tested our molecular approaches for validation on two different TB burden settings: (i) low-burden - 219 clinical isolates of the Hospital Universitario y Politécnico La Fe, Valencia, Spain obtained during the years 2011–2013; and (ii) high-burden - 188 samples from Nigeria Countrywide as well as 78 clinical samples from Liberia. The Nigeria samples were enriched by isolates from cattle lesions.

### Reference collection strains

Samples from Swiss TPH were incubated in liquid media Middlebrook 7H9 (Becton Dickinson) at 37 °C during two weeks, while as those from our laboratory were grown in commercial Middlebrook 7H10 agar (Becton Dickinson) with OADC supplement. In all cases, DNA extraction method was performed following the CTAB method^31^. Purified DNA was used to perform our molecular approaches.

### Sample preparation

In the case of the validation datasets, we tested our molecular approaches in direct supernatants prior to an inactivation step, following the next procedure: all samples were grown in standard Lowenstein-Jensen solid media (BBL, BD) and incubated at 37 °C during 3–4 weeks; next, the inactivation was performed by a heat-kill cycle of 30 min at 95 °C and centrifuged. We used the heat-inactivated supernatant to perform the molecular assays. Moreover, DNA concentration was quantified using PicoGreen® (Molecular Probes) in samples from Valencia, while Qubit fluorometer (ThermoFisher Scientific) was used on those from West African samples.

### SNP selection for molecular assays

For real-time PCR-HRM method, we used a combination of specific-lineage and sub-lineages SNPs previously described^5,6,14,32,33^ as well as new seven markers developed in the present study. Novel markers were identified by analyzing 34,167 SNPs previously identified in a dataset of 219 globally representative genomes^3^. Given that the vast majority of SNPs in MTBC are billelic and does not show evidence of convergent evolution we applied a parsimony-based approach to map and extract all the specific lineages and sub-lineages SNPs from a global MTBC phylogeny. We used the “Trace Character History” module implemented in MESQUITE (http://www.mesquiteproject.org) to obtain the polymorphisms that were common to each lineage and sub-lineage. We obtained a SNPs candidate list of 2,056 and 1,337 for lineages and sub-lineages, respectively. To choose a SNP candidate for the real-time PCR-HRM, we prioritized synonymous variants detected in essential genes. In all cases, we met this criteria, except in SNP markers for L5 and L4.1.3, in which both were non-synonymous changes. As a proof of concept, all the selected SNPs were validated against a database of 4,595 genomes recently published by our group to assure their stability as markers^20^.

The same SNP dataset was used to identify genomic regions with less than 1 Kb that contain the higher number lineage-specific markers. After mapping the genomic coordinates against H37Rv reference genome (NCBI reference number NC_000962.3), we found two candidate regions harbouring three lineage specific variants each. The first region contained the specific SNPs for L1, L2 and L3, the second region had the diagnostic variants for L3,L4 and L5.Afterwards, we designed an amplification assay including the SNPs of interest in both regions for follow-up Sanger sequencing (Fig. 1).

### Primers design and specificity

All primers for both molecular approaches were specifically designed in this project, except diagnostic SNPs for L4 and L6 that were previously published^5,14^. Oligosequences were designed using the Primer3Plus^34^ online tool (www.primer3plus.com). To make multiplex amplifications in a single tube, we increased the melting temperature by adding AA/TT tail-bases in two primer paired sequences. After primer design, we performed a BLAST^35^ search to evaluate *in silico* specificity. Furthermore, we did conventional PCR to corroborate the size and specificity of the amplicons. In all cases, we used the manufacturer conditions of the KAPA2G Fast PCR amplification kit (KAPABiosystems) adding 500 nM of each forward and reverse primer and 10 ng of DNA in a final volume of 25 μl. PCR products were visualized using standard agarose gel electrophoresis (1.4%) for 1 hr at 110 V. Data containing the diagnostic SNP positions, as well as the primer sequences used for all molecular approaches are in Tables 1–2 (real-time PCR-HRM approaches), and Supplementary Table 1 (Sanger sequencing approach).

### Real-time PCR-HRM for lineages and sub-lineages

We optimized the use of the real-time PCR-HRM technology for the rapid detection of specific MTBC lineages (from L1 to L6, and *M. bovis* clade), as well as L4 sub-lineages. For the most common lineages, we designed two multiplex reactions: one containing the specific oligonucleotides for L2, L3 and L4 and the second including the primers for specific L1 and L6. In contrast, uniplex reactions were developed for L5 and *M. bovis* identification. In all cases, each PCR reaction had a final volume of 10 μl. This reaction consisted in 5 μl of the KAPA HRM FAST PCR Master Mix (KAPABiosystems), which includes the unspecific EvaGreen® dye and dNTPs; 2.5 mM of MgCl_2_ (25 mM) and 10 ng of template DNA. Every forward and reverse primers had different concentrations depending on the multiplex reaction. In the first multiplex reaction, we added 200 nM of each primer for L2 and L3, while for L4, we used 600 nM of each oligonucleotide. In the second multiplex reaction, the concentration of each pair of primers was 200 nM. In the case of the Uniplex assay (L5 and *M.bovis*), a total of 400 nM of each primer were added to the mix. Finally, distilled water was added to complete a 10 μl final volume. For all lineages the PCR amplification step consisted in an initial denaturation step at 95 °C for 5 min; 40 cycles of denaturation (95 °C, 10 s), annealing (57 °C, 20 s) and final extension (72 °C, 20 s).

Real-Time PCR-HRM for L4 specific sub-lineages was performed following uniplex reaction conditions described above, with the exception of the annealing step, which was 55 °C due to the oligonucleotides melting temperature. All reactions were performed in three technical replicates per sample to test the reproducibility of the assay.

The HRM assay was performed at the end of each reaction and consisted of one cycle of increasing temperature from 45 °C to 97 °C at ramps rate of 2.2 °C/s. Fluorescence signal changes were collected at the end for posterior analysis. In every run we used several controls. A free-DNA well serve as a non-template control. In addition, reference DNAs for all lineages were used to assign the melting curve of the sample to one for the lineage/sub-lineage and as controls.

### Analysis of the melting curves in HRM assay

All the real-time PCRs (multiplex and uniplex) reactions and HRM curves analyses were performed with a Roche LightCycler480 instrument (Roche Applied Science, Germany) and Gene Scanning software, respectively. This analysis consists of four steps: (1) identify samples that did not amplify as negatives to exclude them from the analysis; (2) normalize the melting curve data indicating the values of initial and final signal fluorescence for all samples; (3) adjust the temperature of the normalized melting curves at the point where the DNA is denatured to distinguish the changes in the shape of samples; and (4) finally, the melting curves are represented by a difference plot. The program allows selecting a baseline curve to show the differences based on this. In all the cases negative controls for the lineage or sub-lineages evaluated were used for this baseline curve.

### Sanger sequencing

We performed Sanger sequencing on the reference set to identify major lineages. First, the amplification step was carried out with conventional PCR using the same reaction conditions as described above (primers specificity section). Then, PCR products were labeled and purified according to the BigDye Terminator v3.1 Cycle Sequencing Kit (AppliedBiosystems) and PCR ExcelaPure 96-Well UF Purification Kit (EdgeBioSystems) protocols, respectively. The two target regions were sequenced with the ABI 3037xl DNA analyzer (AppliedBiosystems). Finally, the resulting sequences were analyzed using Pregap5 and Gap5 programs^36^, both included in the Staden package.

### Performance of the molecular techniques

We evaluate the performance of the techniques by calculating the accuracy, sensitivity, specificity, positive predictive value and negative predictive value of each. WGS lineage definition was used as a gold standard genotyping method. We tested a total of 76 whole-genome sequenced heat-inactivated clinical samples, 43 from Liberia dataset and 33 from Valencia dataset.

### Biosafety procedures

DNA extractions from cultures were done in a BSL-3 facility as per WHO recommendations. Samples from Nigeria and Liberia were heat-inactivated at the Nigeria laboratory. Once arrived, s second inactivation step to assure killing of the bacteria was done at FISABIO’s (Spain) BSL-3 facility. In the case of the Valencia samples, culture procedures as well as heat-inactivated step were performed at the Hospital Universitario Politécnico la Fe BSL-3.

## Supplementary information

Supplementary Material

## Data Availability

The datasets generated during and/or analysed during the current study are available from the corresponding author on reasonable request. The Sanger sequences generated were uploaded to the NCBI database under submission numbers 2262663 and 2262671.
