# Supplementary material for: Development and application of affordable SNP typing approaches to genotype Mycobacterium tuberculosis complex strains in low and high burden countries
Source: Sci Rep. 2019 Oct 25;9:15343. doi: 10.1038/s41598-019-51326-2 (PMC6814786; doi:10.1038/s41598-019-51326-2)
Supplement: Supplementary file 1 — Supplementary Material [file 41598_2019_51326_MOESM1_ESM.pdf]

SUPPLEMENTARY INFORMATION

Development and application of affordable SNP typing to genotype *Mycobacterium tuberculosis* complex strains in low and high burden countries

Irving Cancino-Muñoz, Ana Gil-Brusola, Manuela Torres-Puente, Carla Mariner-Llicer, John Dogba, Victor Akinseye, Kehinde Adesokan, Ayi Kwaghe, Francis Ejeh, Simeon Cadmus‡, and Iñaki Comas ‡, *.

* Corresponding author, Email: icomas@ibv.csic.es

‡ These senior authors contributed equally to this work.

Supplementary Table 1. Specific primers used in Sanger sequencing molecular approach

MTBC Lineage*	SNP position†	Nucleotide change‡	Gene	Primers 5'-3'	Amplicon size	Reference
L1, L2 and L5	L1 4357773	G/A	Rv3878/ Rv3879c	F-ACCCTCAACAACCACAACGT<br>R-CGACACTACCGATCAGCGTT	386bp	This study
	L2 4357804	T/G				
	L5 4357657	G/A				
L3, L4 and L6	L3 1281984	G/A	Rv1155/ intergenic region	F-GATGGTCATACGCCGTTGCT<br>R-CTCTTGCGGGGACTTCGATT	402bp	This study
	L4 1281771	C/T				
	L6 1281685	C/G				

* Nomenclature proposed by Comas et al [1].

† Genomic position on the H37Rv reference genome (NCBI, NC_000962.3).

‡ Allelic change in the reference genome.

Supplementary Table 2. Reference samples used in this study. All isolates were previously characterized by whole-genome sequencing

Sample ID	Source*	MTBC lineage†	LSP lineage‡	Spoligotype familiy§
N0067	Swiss TPH	L1	Indo-Oceanic	EAI
N0153	Swiss TPH	L1	Indo-Oceanic	EAI
N1068	Swiss TPH	L1	Indo-Oceanic	EAI
N0053	Swiss TPH	L2	East-Asian	Beijing
N0150	Swiss TPH	L2	East-Asian	Beijing
N1007	Swiss TPH	L3	East-African-Indian	CAS
N1022	Swiss TPH	L3	East-African-Indian	CAS
N1057	Swiss TPH	L4.1.1	Euro-American	X
N0148	Swiss TPH	L4.1.1	Euro-American	X
N0142	Swiss TPH	L4.1.1	Euro-American	X
G02	This study	L4.1.2	Euro-American	Haarlem
G287	This study	L4.1.2	Euro-American	Haarlem
G1010	This study	L4.1.2	Euro-American	Haarlem
N1204	Swiss TPH	L4.1.3	Euro-American	Ghana
G770	This study	L4.1.3	Euro-American	Ghana
N1263	Swiss TPH	L4.2	Euro-American	
G440	This study	L4.2	Euro-American	
G551	This study	L4.2	Euro-American	
G450	This study	L4.3	Euro-American	LAM
G186	This study	L4.3	Euro-American	LAM
G1068	This study	L4.3	Euro-American	LAM
G200	This study	L4.4	Euro-American	
G564	This study	L4.4	Euro-American	
N0163	Swiss TPH	L4.5	Euro-American	
N1277	Swiss TPH	L4.6.1	Euro-American	Uganda
N1207	Swiss TPH	L4.6.2	Euro-American	Cameroon
G630	This study	L4.6.2	Euro-American	Cameroon
G818	This study	L4.6.2	Euro-American	Cameroon
N1770	Swiss TPH	L4.10	Euro-American	
G109	This study	L4.10	Euro-American	
G280	This study	L4.10	Euro-American	
N1176	Swiss TPH	L5	West-African-1	AFRI2
N1063	Swiss TPH	L5	West-African-1	AFRI2
N1272	Swiss TPH	L5	West-African-1	AFRI2
N0091	Swiss TPH	L6	West-African-2	AFRI1
N1202	Swiss TPH	L6	West-African-2	AFRI1
N1177	Swiss TPH	L6	West-African-2	AFRI1
G578	This study	*M.bovis*	*M.bovis*	*M.bovis*
G513	This study	*M.bovis*	*M.bovis*	*M.bovis*
G1020	This study	*M.bovis*	*M.bovis*	*M.bovis*

* Refers to the sample origin, Swiss TPH means Swiss Tropical Public Health Institute, and This study refers that the sample were obtained by a local ongoing population study.
† Nomenclature proposed by Comas et al and Stucki et al [1,2].
‡ Lineage name proposed by Gagneux et al [3].
§ Nomenclature proposed by Demay et al [4].
Abbreviations: LSP, Long Sequence Polymorphisms; TPH, Tropical Health Institute.

Supplementary Table 2. Specific primers used in Sanger sequencing molecular approach

MTBC Lineage*	SNP position†	Nucleotide change‡	Gene	Primers 5'-3'	Amplicon size	Reference
L1, L2 and L5	L1 4357773	G/A	Rv3878/ Rv3879c	F-ACCCTCAACAACCACAACGT R-CGACACTACCGATCAGCGTT	386bp	This study
	L2 4357804	T/G				
	L5 4357657	G/A				
L3, L4 and L6	L3 1281984	G/A	Rv1155/ intergenic region	F-GATGGTCATACGCCGTTGCT R-CTCTTGCGGGGACTTCGATT	402bp	This study
	L4 1281771	C/T				
	L6 1281685	C/G				

* Nomenclature proposed by Comas et al [1].

† Genomic position on the H37Rv reference genome (NCBI, NC_000962.3).

‡ Allelic change in the reference genome.

Supplementary Figure 1. PCR products for the Sanger sequencing molecular assay (part 1). The figure shows the amplified products for Region 1 (specific markers for L1, L2 and L5). Three samples for each lineage were tested, except for L5, which only had 2 samples (wells 14-15). The amplicon size is 386bp. Well 19 was DNA-free and was considered as a negative control. The molecular weight-size ranged from 250-10,000bp.

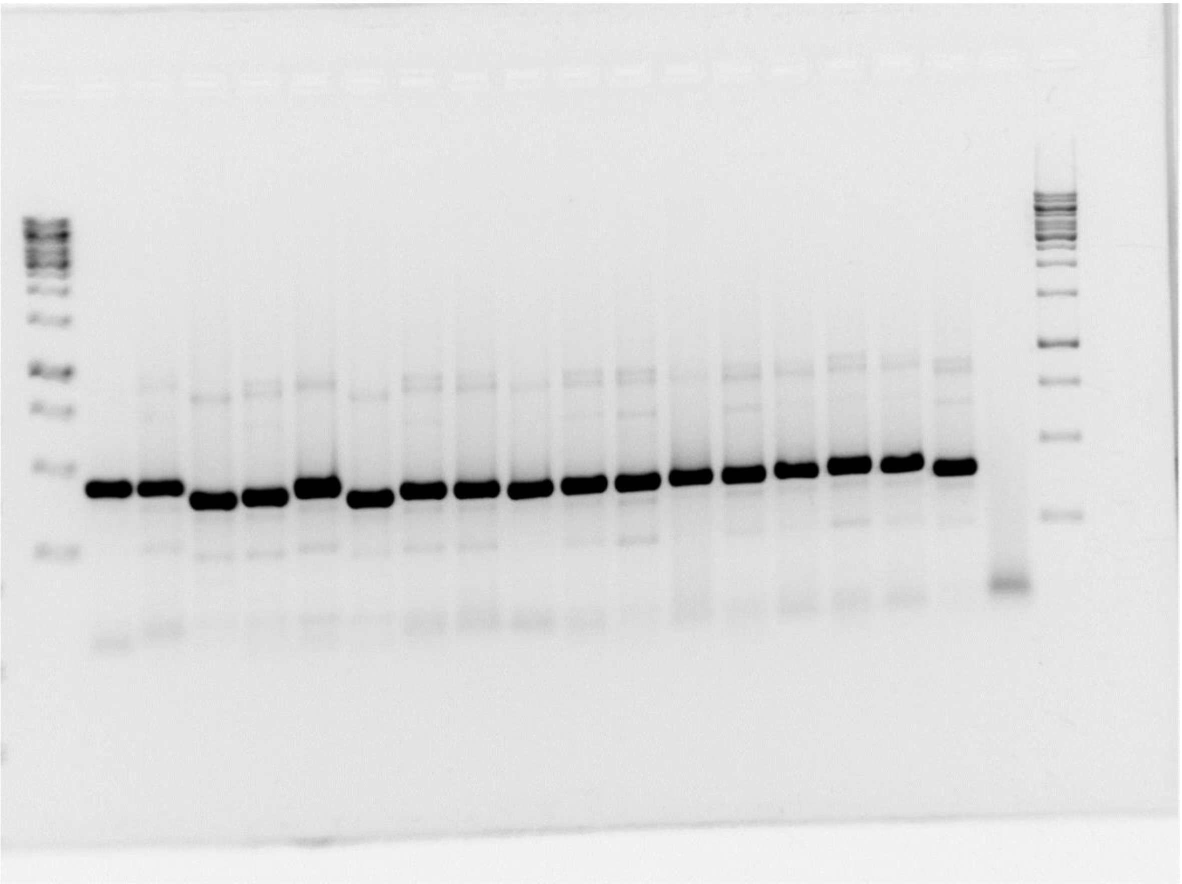

Supplementary Figure 2. PCR products for the Sanger sequencing molecular assay (part 2). The figure shows the amplified products for Region 1 (specific markers for L3, L4 and L6). Three samples for each lineage were tested, except for L5, which only had 2 samples (wells 14-15). The amplicon size is 402bp. Well 19 was DNA-free and was considered as a negative control. The molecular weight-size ranged from 250-10,000bp.

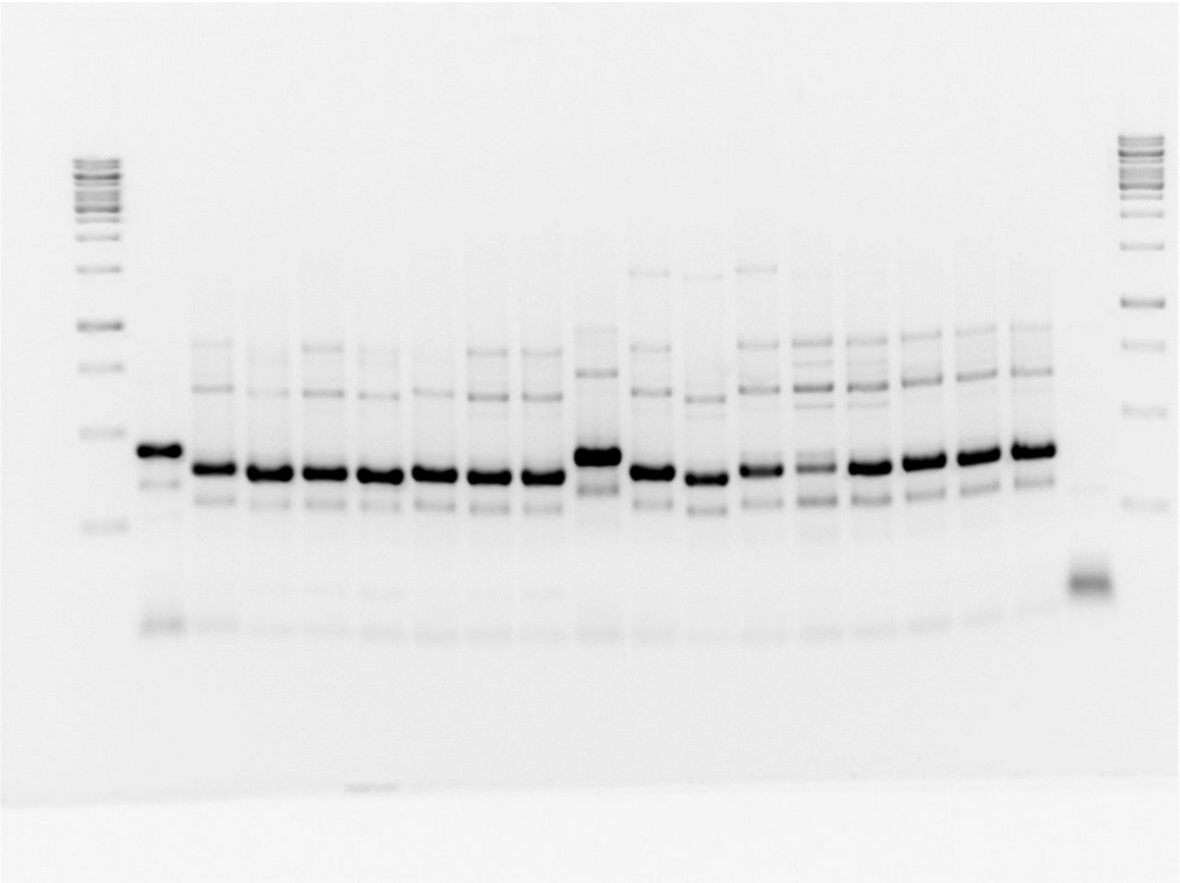

Supplementary Figure 3. **Sanger sequencing amplified Region 1 to identify lineage 5**. The chromatograms shows the specific region that contains the lineage 5 diagnostic SNP for different samples. The specific polymorphism is marked in blue. An adenine (represented in green) is detected instead of a guanine (represented in black) at the position 169. In this case, the sample N0135 harbors the diagnostic marker for lineage 5, while the rest presented the wild-type allele.

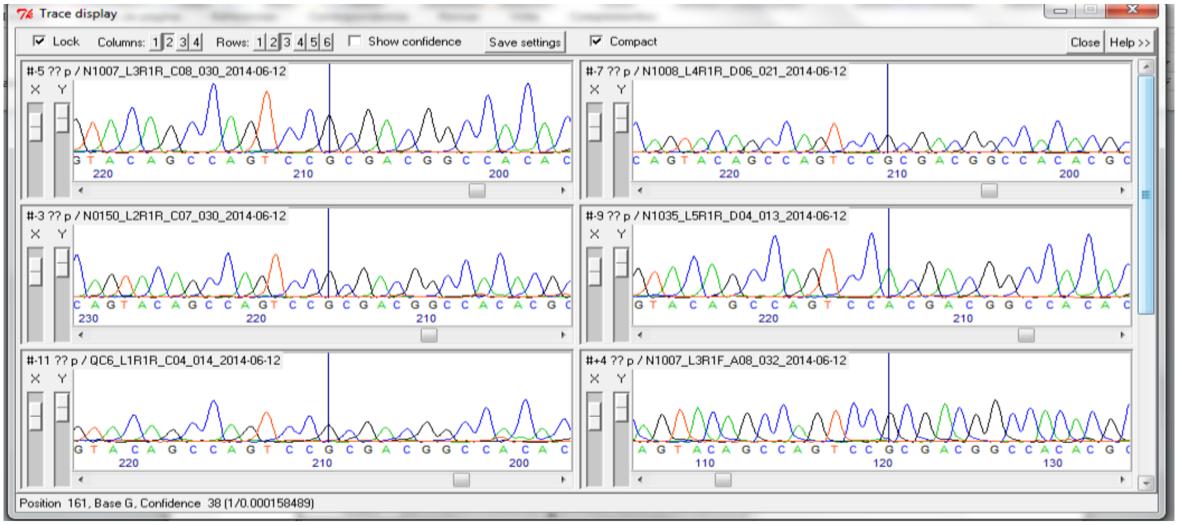

SUPPLEMENTARY REFERENCES

1. Comas, I. *et al.* Human T cell epitopes of *Mycobacterium tuberculosis* are evolutionarily hyperconserved. *Nat. Genet.* **42**, 498–503 (2010).
2. Stucki, D. *et al. Mycobacterium tuberculosis* lineage 4 comprises globally distributed and geographically restricted sublineages. *Nat. Genet.* **48**, 1535–1543 (2016).
3. Gagneux, S. *et al.* Variable host-pathogen compatibility in *Mycobacterium tuberculosis*. *Proc. Natl. Acad. Sci.* **103**, 2869–2873 (2006).
4. Demay, C. *et al.* SITVITWEB – A publicly available international multimarker database for studying *Mycobacterium tuberculosis* genetic diversity and molecular epidemiology. *Infect. Genet. Evol.* **12**, 755–766 (2012).
